# Eliciting false insights with semantic priming

**DOI:** 10.3758/s13423-021-02049-x

**Published:** 2022-02-02

**Authors:** Hilary Grimmer, Ruben Laukkonen, Jason Tangen, William von Hippel

**Affiliations:** 1 School of Psychology, The University of Queensland, Brisbane, QLD Australia; 2 Cognitive Psychology, Vrije Universiteit Amsterdam, Amsterdam, The Netherlands

**Keywords:** Insight, Problem solving, ‘Aha’ experience, Phenomenology

## Abstract

The insight experience (or ‘Aha moment’) generally evokes strong feelings of certainty and confidence. An ‘Aha’ experience for a false idea could underlie many false beliefs and delusions. However, for as long as insight experiences have been studied, false insights have remained difficult to elicit experimentally. That difficulty, in turn, highlights the fact that we know little about what causes people to experience a false insight. Across two experiments (total *N* = 300), we developed and tested a new paradigm to elicit false insights. In Experiment 1 we used a combination of semantic priming and visual similarity to elicit feelings of insight for *incorrect* solutions to anagrams. These false insights were relatively common but were experienced as weaker than correct ones. In Experiment 2 we replicated the findings of Experiment 1 and found that semantic priming and visual similarity interacted to produce false insights. These studies highlight the importance of misleading semantic processing and the feasibility of the solution in the generation of false insights.

**Supplementary Information:**

The online version contains supplementary material available at 10.3758/s13423-021-02049-x.

## Introduction

The ‘Aha’ experience is not only exciting, it is also informative; people’s self-reported insights consistently signal the accuracy of their solutions (Danek et al., 2016; Danek & Wiley, 2017; Hedne et al., 2016; Salvi et al., 2016; Webb et al., 2016, 2018). Despite the strength and reliability of this relationship, the feeling of insight does not guarantee that a solution will be correct. Indeed, people have experienced ‘Aha’ moments for incorrect solutions (Danek et al., 2016; Danek & Wiley, 2017; Valueva et al., 2016; Webb et al., 2016). These so-called *false insights* are difficult to investigate because they have not been evoked experimentally. As a consequence, little is known about their causes. In this paper, we introduce a new experimental paradigm to induce false insights and explore their origins.

Insight moments are important for several reasons: they mark important achievements (Irvine, 2015; Ovington et al., 2018), they are highly memorable (Danek & Wiley, 2020), and they facilitate learning (Kizilirmak et al., 2016). Research has unraveled cognitive processes that underlie insights (Ohlsson, 1984) and more recently has developed phenomenological measures of the insight experience that enable its investigation on a case-by-case basis (Bowden & Jung-Beeman, 2007). These studies reveal that ‘Aha’ moments are accompanied by strong feelings of surprise, positive affect, and certainty (Aziz-Zadeh et al., 2009; Bowden et al., 2005; Danek et al., 2014b; Kounios & Beeman, 2009; Subramaniam et al., 2008). Perhaps most importantly, the heightened confidence associated with an insight experience gives problem-solvers the impression that they have discovered something objectively true (Danek et al., 2014a, 2020; Metcalfe & Wiebe, 1987; Topolinski & Reber, 2010).

Many famous ‘Aha’ moments took considerable time to prove they were accurate, yet problem-solvers often describe a sense of spontaneous certainty without clear evidence. For example, mathematician Yitang Zhang took months to prove his solution to the twin prime conjecture, yet described his moment of insight by saying, “I immediately realized that it would work” (Klarreich, 2013). Experimental evidence that insight moments enhance certainty can be found in research by Laukkonen et al. (2020), who showed that people were more likely to judge statements as true when the statement contained an irrelevant anagram that elicited an ‘Aha’ moment. In a similar paradigm, Dougal and Schooler (2007) also found that successfully solved anagrams were recalled more frequently on a subsequent memory task than solutions to unsolved anagrams, and that this effect diminished when a delay between anagram solving and the memory task was introduced. These findings suggest that insight phenomenology is so closely tied to our judgments of truth that we can misattribute the feelings of certainty to a temporally contiguous, yet conceptually irrelevant, stimulus and mistake feelings of solving for feelings of remembering.

There are several theoretical accounts about why ‘Aha’ moments tend to be correct (Danek & Salvi, 2020; Laukkonen et al., 2018; Salvi et al., 2016), but underlying all of them is the idea that people can *feel* that they have suddenly solved a problem after experiencing an impasse. As with other feelings, the feeling of insight may not always be accurate, but few studies have directly addressed false insights. One of the only studies comparing false and true insights was conducted by Danek and Wiley (2017), who asked participants to figure out a series of magic tricks. Participants rated any ‘Aha’ experiences in terms of how strong their feelings of surprise, pleasure, satisfaction, and confidence were. The authors found that false ‘Aha’ moments, although uncommon, were rated lower on surprise, pleasure, satisfaction, and confidence than true ‘Aha’ moments.

Early research on insight moments only examined correctly solved problems and distinguished between those solved with and without an ‘Aha’ experience (e.g., Danek et al., 2016; Jung-Beeman et al., 2004; Webb et al., 2016). This practice made it difficult to demonstrate how frequent false insight are, and how they differ from correct insights. By increasing the rates of false solutions, we could also increase the chances for false insights to occur, allowing us to investigate the relationship between ‘Aha’ intensity and accuracy in an experimentally valid and efficient manner, providing a window into their origins and offering information about the processes that generate them.

Although false insights have never been generated through experimental manipulation, there is an analogous – and potentially informative – line of experiments on the creation of false memories (Gallo, 2010). The most famous example is the semantic priming paradigm re-introduced by Roediger and McDermott (1995); see also Deese, 1959). In this paradigm (known as the DRM paradigm), participants are given a list of study words, all of which are related to the same semantic category (e.g., bed, rest, tired, dream), and are then tested for their memory of the study list. Critically, the memory test contains one word that was not present on the study list but is closely related to the semantic category (e.g., sleep). Roediger and McDermott (1995) found that people falsely (and confidently) remembered the related target word as having been presented.

Semantic priming has been widely used across a number of tasks and settings. For example, people are faster to solve anagrams related to semantically primed compared to unprimed categories (Schuberth et al., 1979; White, 1988). In combination, these lines of research suggest that semantic priming can lead people toward both correct and incorrect solutions. Because semantic priming makes certain words more accessible, we reasoned that priming could also make people more likely to mistakenly solve an anagram with a semantically primed associate. Thus, we predicted that solving anagrams after being primed with misleading semantic information could lead participants to have ‘Aha’ experiences for anagram solutions that are objectively incorrect (i.e., elicit false insights). The goal of the current research was to test this possibility, and thereby obtain a better understanding of the mechanisms underlying false insights.

## Experiment 1

In Experiment 1 we elicited false insights by priming participants with a list of semantically related words and then presenting a series of four anagrams each relating to the study list in a different way. The anagrams were either made from words (1) chosen at random, (2) presented on the study list, (3) not presented but semantically associated with the list, or (4) visually similar (differing by one or two letters) to an unpresented but semantically associated word. We predicted that people would be lured into having more false insights when solving this final category of visually misleading anagrams that resemble a primed concept compared to the other kinds of anagrams. We also predicted that the phenomenological intensity of false insights would be lower than correct insights, regardless of the type of anagram that led to them. Finally, we expected that participants who experienced more false insights for the deceptive lure anagrams would also be more likely to falsely remember these incorrect solutions as having appeared on the study list.

### Method

#### Open practice statement

This experiment is preregistered on the Open Science Framework. The data, materials, video instructions, experimental design, exclusion criteria, and analysis scripts are available at: https://osf.io/nu3mr/?view_only = c09eedcf8c4545b9a834be405fee90ec

#### Participants

One hundred and fifty undergraduate psychology students (99 females, mean age = 22.35 years) from The University of X took part in the experiment and were awarded partial course credit for their time. Based on Danek and Wiley (2017), we anticipated a moderate effect size, and established that 150 participants would provide sufficient sensitivity (power = .84) to detect an effect size of *d* = 0.45.

#### Design and materials

**Fig. 1:**
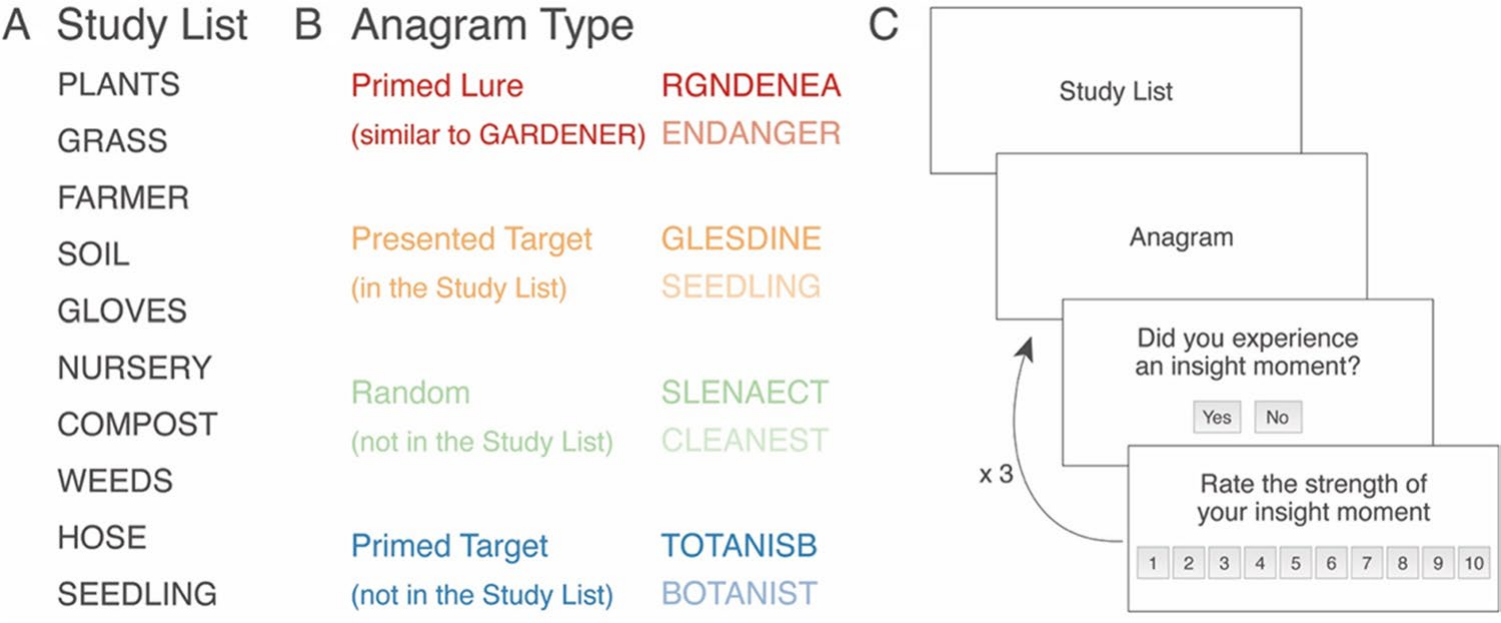
Example study list, anagrams, and experiment flow of a single trial

We generated pairs of similar-looking words (i.e., words of a similar length that share most of their letters), and we then generated lists of ten associated words for each word in the pair. For example, the word pair GARDENER and ENDANGER share most of their letters and are the same length. We then created a list of ten words that were semantically associated with the word GARDENER (e.g., FLOWERPOT, SHOVEL, SEEDLING, etc.) and ten words associated with the word ENDANGER (e.g., HAZARD, THREATEN, RISK). Through this process, we generated six pairs of similar-looking words along with ten semantically associated words for each word in the pair, resulting in six pairs of word lists. One list from each pair and its associated anagrams were put into two counterbalanced versions of the experiment. For example, half of the participants saw the words that primed gardener. This process allowed us to eliminate any effects that might be a function of the specific stimuli rather than the combination of the primes and visual similarity. We randomly allocated half of the participants to perform one of the two versions of the counterbalanced stimuli. Participants thus read one list from each pair (see Fig. 1a) and were then presented with four different anagrams (see Fig. 1b). These anagrams each served a different purpose in terms of our hypotheses. One anagram was a scrambled word from the priming list, which we refer to as the *presented target.*^1^ Another anagram, the *primed target*, was not presented on the list, but was semantically associated with the words from the list. The critical anagram, which we called the *primed lure*, was visually similar to a word that was semantically related to the studied list of words, but in fact was really an anagram for a semantically unrelated word that was not presented in the priming list*.* Finally, we included a random word as a control item that was neither primed nor semantically related (see Fig. 1b). This experiment thus followed a mixed design, with counterbalancing condition as a between-subjects factor, and anagram type as a within-subjects factor.

After we made these lists for all our original word pairs, we used the word-frequency database SUBTLEX-UK (van Heuven, Mandera, Keuleers, & Brysbaert, 2014) to ensure that our words were common enough to assume that participants would be familiar with them. This database of 160,022 words was created by collecting the subtitles from nine British TV channels over a 3-year period and assigning each word a Zipf value to indicate its relative frequency. The Zipf scores can range from 1 (very low frequency) to 6 (very high frequency words). We obtained the Zipf scores for each word on the study lists, with the goal of using words with a value greater than 3 (which van Heuven et al., 2014, propose as the tipping point from low- to high-frequency words). For the control word in the anagram task, we averaged the Zipf scores of the three chosen anagram words, and using that average, we selected a random word of the same length with a Zipf score equal to that average.

To generate anagrams that looked optimally similar to the intended solution, using MATLAB, we generated every possible scrambled configuration of our four anagram word pairs that ranged from most similar to least similar to the intended solution and computed the cosine similarity among the pixels of each scrambled word (see Vokey & Jamieson, 2014). The cosine value of two images indicates how close they are in multidimensional image space and thus how visually similar they are to one another (see OSF for Matlab script). For the primed lures, cosine values closer to 1 suggest that the scrambled word is visually similar to the intended solution – not the actual solution. For the other anagram types, cosine values closer to 1 suggest that they are visually similar to their correct unscrambled solution. Thus, we created the primed lures by entering the *intended* solution as the target and scrambling the lure (the unrelated but visually similar word) to resemble the intended solution at the ideal level of similarity. The three control anagrams were simply created by scrambling the word itself – the actual solution – to the ideal level of visual similarity. After informal pretesting, we chose anagrams with cosine values of 0.85 to ensure the anagrams were not too dissimilar from their intended solution (and therefore unsolvable), but not so similar that participants might solve them without feelings of impasse and subsequent ‘Aha’ experiences upon resolution. The experiment was programmed using LiveCode and presented to individual participants on laptops.

#### Measures and procedure

Testing took place in a room with four laptops. After obtaining verbal consent, each participant sat at a computer and played an instruction video that explained how each trial of the experiment would be conducted. The instructions stated that the task was to remember as many words from the study list as possible and recall them after performing an anagram task. Each trial began with participants studying a list of ten semantically associated words, which were presented one at a time on the screen and spoken aloud by the computer voice through the headphones. After the list was completed, participants were instructed to press the spacebar once they were ready to solve the anagrams. The four anagrams described above were then presented in a random order, and participants were told to press the spacebar once they had thought of a solution. There was no time limit for solving the anagrams, but participants were encouraged to work quickly and attempt every anagram. Participants’ reaction time was also recorded in milliseconds for each trial. The full transcript of these instructions is provided in Appendix 1. Upon pressing the spacebar, the anagram disappeared from the screen, and participants were instructed to type their solution into a box on the screen (see Fig. 1c).

After entering each anagram solution, participants were prompted to indicate whether they experienced an ‘Aha’ moment or not (Laukkonen & Tangen, 2018). If participants reported having an ‘Aha’ moment, they were asked to rate the intensity of their ‘Aha’ experience on a scale from 1 (“very weak’) to 10 (‘very strong’). After solving all four anagrams, they were prompted to type all the words they could recall from the study list before proceeding to the next trial. The memory task was used to investigate whether participants who had false insights for the primed lures also falsely remembered these lures as appearing on the study list. False memories were thus recorded when participants included the incorrect solution primed by the lure (i.e., the primed lure) on their recall list at the end of each trial. This process was repeated six times. For additional measures and analyses not included in this paper, see the Online Supplementary Materials (OSM).

### Results

**Fig. 2:**
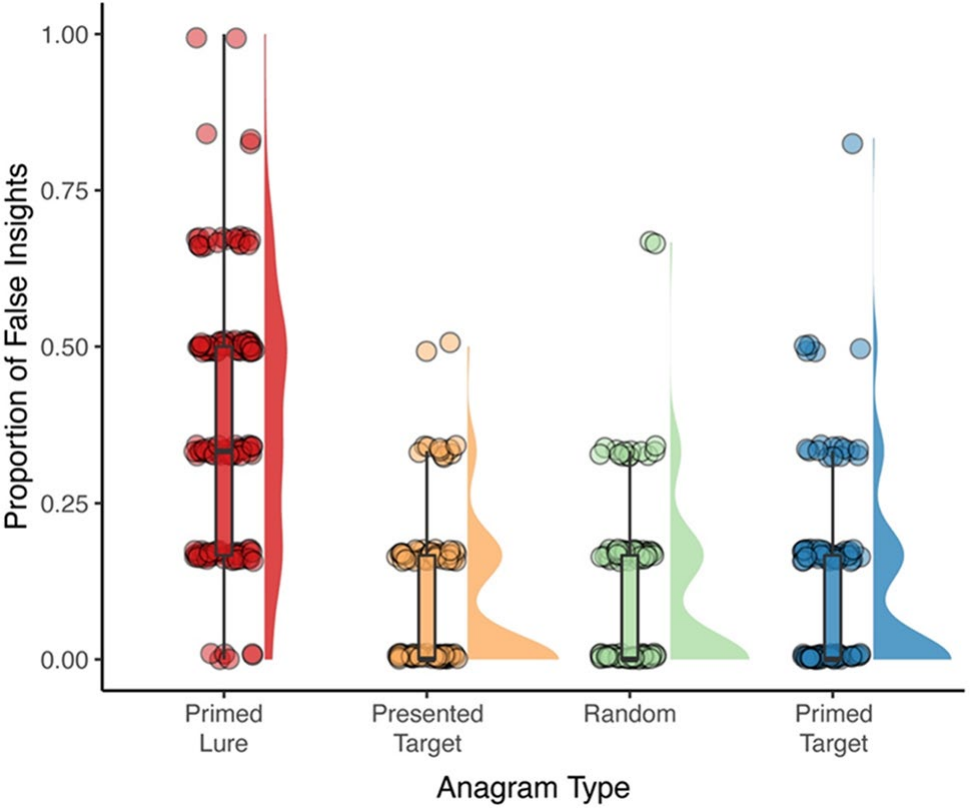
Percentage of trials with false insights reported for each type of anagram

The average solution time and correct solution rates for each anagram type and counterbalancing conditions are presented in Table 1. We computed the proportion of all trials for each anagram type with reported false insights as the number of incorrect anagram solutions accompanied by an ‘Aha’ moment divided by the number of trials for each anagram type. The ‘raincloud’ plots in Fig. 2 depict the proportion of trials with false insights across the four anagram

**Table 1:** Mean reaction time in seconds, proportion of correctly solved trials, and proportion of trials with false insights reported for each anagram type in each counterbalancing condition

	Counterbalancing condition	Primed lure	Presented target	Random	Primed target
Mean reaction time (*SD*)	A (*N* = 73)	31.20 (20.24)	34.99 (22.03)	49.32 (26.26)	24.83 (17.90)
	B (*N* = 77)	41.07 (20.44)	26.79 (17.10)	36.36 (18.50)	25.80 (19.47)
Proportion correct	A (*N* = 73)	0.27 (0.15)	0.67 (0.26)	0.37 (0.23)	0.47 (0.28)
	B (*N* = 77)	0.04 (0.08)	0.67 (0.21)	0.52 (0.22)	0.58 (0.23)
Proportion of trials with false insights	A (*N* = 73)	0.35 (0.25)	0.09 (0.15)	0.09 (0.14)	0.08 (0.13
	B (*N* = 77)	0.39 (0.19)	0.09 (0.14)	0.08 (0.11)	0.06 (0.10)

types, combining boxplots, raw jittered data, and a split-half violin. This figure shows that the primed lure anagrams produced the highest rates of false insights out of the four anagram types.

To test our prediction that the primed lures would elicit more false insights than the other three anagram types, we ran a mixed ANOVA on the proportion of trials with false insights for each anagram type with counterbalancing condition as a between-subjects factor.^2^ This analysis revealed no significant difference between the two counterbalancing conditions, suggesting that the effect of anagram type was the same for both sets of stimuli, *F*(1,148) = 0.04, *p* = .943, η^2^_G_ < .001. As predicted, a significant difference between the number of false insights elicited by each anagram type emerged, *F*(3,444) 171.52, *p* < .001, η^2^_G_ = .39. We tested our planned comparisons using post hoc Tukey *t*-tests. As predicted, these revealed that the primed lure anagrams (*M =* 0.37, *SD =* 0.22) elicited significantly more false insights than the presented target (*M =* 0.07, *SD = 0.11*; *t*(444) = 19.17, *p* < .001, *d* = -1.73, *CI* = 0.26, 0.34), the primed target (*M =* 0.09, *SD =* 0.15; *t*(444) *=* 17.83, *p* < .001, *d* = -1.49, *CI* = 0.24, 0.32), and the random anagrams (*M =* 0.08, *SD* = 0.13*; t*(444) *=* 18.47, *p* < .001, *d* = -1.61, *CI* = 0.25, 0.33).

To compare the intensity of true and false insights, we looked at only trials on which an insight moment was reported, and scored them as either correct or incorrect. We then selected participants who reported at least one false and one correct insight (*N* = 142) and computed the mean intensity ratings given to false and correct insights for each participant across all anagram types. Because we were interested in the phenomenological difference between false and correct insights, we included all false insights in this analysis, regardless of the type of anagrams on which they occurred – although the majority were for primed lure anagrams (60.35%). A paired *t*-test revealed that false insights were rated as significantly less intense (*M* = 5.81, *SD* = 1.93) than correct insights (*M* = 6.12, *SD* = 1.74 *t*(141) = 2.57, *p* = .011, *d =* -0.22, *CI* = 0.07, 0.54. The correlation between accuracy and insight intensity was also significant, *r* = .42, *p*<.001, *CI* = .28, .54. To test whether false insights predicted false memories, we examined the correlation between participants’ total false insights for primed lures and their total number of false memories for primed lures in the recall task. We considered only primed lures for this analysis to ensure the opportunities for false memory as we defined above (primed lures being reported on the recall list) matched the opportunities for false insights. This relationship was positive and significant, *r* = .18, *p* = .029, *CI* = .02, .33, such that participants who experienced more false insights in the primed lure condition also falsely recalled more primed lures.

## Experiment 2

The goal of Experiment 2 was to replicate the findings of Experiment 1 and assess the degree to which the false insight effect in Experiment 1 was driven by either the semantic priming or the misleading visual configuration of the anagrams. We predicted that participants who saw both semantic priming and visually similar anagrams would experience the highest proportion of false insights (as in Experiment 1), followed by participants exposed to semantic priming and given randomly scrambled anagrams, followed by participants who were not semantically primed but were given visually similar anagrams. Thus, we expected that the false insight effect documented in this experiment would be driven more by semantic priming than visual similarity. Finally, we expected that participants would again report lower subjective intensity for false versus correct insights. We did not pursue the relationship between false insights and false memories in this experiment as the aim of this study was to understand the driving factors of the false insight effect.

### Method

#### Open practice statement

This experiment is preregistered on the Open Science Framework. The data, materials, intended study design, exclusion criteria, and analysis scripts are available at https://osf.io/ez4y6/?view_only = 97a1183e9f954b749f41aab3ce0424bf.

#### Participants

Given the mean differences between each anagram type observed in Experiment 1, we simulated and analyzed the results from 2,000 datasets based on 37 participants in each of the four groups. This sensitivity analysis revealed that our design would be sufficiently powered to detect an effect size of η^2^_G_ = .15 for the main effect of Anagram Type in all 2,000 of these simulations (100%). By decreasing the mean differences between the four anagram types to derive the smallest effect size of interest, which was η^2^_G_ = .02 (Lakens et al., 2018), we could still detect a significant main effect in 1,600 out of 2,000 simulated datasets (80%). This entire sensitivity analysis is documented at (https://osf.io/ez4y6/files/). We therefore decided to use the same sample size as Experiment 1. A sample of 150 native-English speaking participants (79 female, 66 male, four other) with a mean age of 29.67 years was recruited using the online crowdsourcing platform Prolific Academic, who received $6 for their participation.

#### Design and materials

This experiment had a 2 (Semantic Priming: present, absent) 𝗑 2 (Visual Similarity: present, absent) 𝗑 4 (Anagram Type: primed lure, presented target, random, primed target) mixed design with Semantic Priming and Visual Similarity as between-subjects factors, and Anagram Type as a within-subjects factor. Since there was no difference in the rates of false insights produced by each counterbalancing condition in Experiment 1, we included both sets of stimuli in Experiment 2 but presented them randomly to participants so they were not included as a factor in our analyses. The Semantic Priming and Visual Similarity materials were the same as the first experiment. For the conditions without semantic priming, we presented lists of randomly generated words (created by https://randomwordgenerator.com) instead of the semantic associates lists. In these conditions, the anagrams were the same visually similar configurations (cosine of .85) used in Experiment 1 but lacked any semantic relation to the priming list. Because we expected the effect of anagram type to depend on the presence of semantic priming, these conditions served as controls to assess whether false insights would occur less frequently for the primed lure anagrams when there was no semantic relationship to the study list, despite being in a misleading configuration. For the conditions without visual similarity, we used the same priming lists as in Experiment 1 but did not compute a cosine similarity for any of the anagrams relative to their intended solution. Rather, for the three control anagrams, we scrambled the words using an online random word scrambling tool (instead of arranging them to resemble the correct solution at a.85 level of cosine similarity). Likewise with the primed lures, instead of arranging the incorrect solution to resemble a specific primed associate, we randomly scrambled it using the same tool. This process thereby removed the effect of visual similarity to investigate the possible interaction between semantic priming and anagram types. We expected this manipulation to demonstrate that regardless of how the anagrams were scrambled, primed lures would elicit false insights more than other anagram types simply due to their semantic association with the study list.

We programmed the experiment to run as closely as possible to Experiment 1, with each word being presented at the same rate, and the answer boxes appearing for the same time and in the same fashion. One difference was that the word lists were not spoken aloud by the computer, but simply appeared on the screen instead.

#### Measures and procedure

The procedure was nearly identical to Experiment 1 except participants provided their consent electronically. Due to the deviations from the original experiment necessitated by the online format, participants received written instructions instead of the video used in the first experiment. A full transcript of these instructions is available in Appendix 1.

### Results

As in Experiment 1, we isolated trials with reported insight moments and computed the proportions of false insights for each condition Table 2.

**Table 2:** Mean reaction time in seconds, proportion of correctly solved trials, and proportion of trials with false insights reported for each anagram type in each counterbalancing condition

	Semantic priming	Visual similarity	Primed lure	Presented target	Random	Primed target
Proportion of trials with false insights	Present	Present (*N* = 41)	0.24 (0.16)	0.02 (0.08)	0.02 (0.07)	0.02 (0.06)
		Absent (*N* = 32)	0.12 (0.16)	0.02 (0.09)	0.01 (0.04)	0.02 (0.09
	Absent	Present (*N* = 36)	0.07 (0.18)	0.03 (0.12)	0.03 (0.17)	0.05 (0.18
		Absent (*N* = 41)	0.09 (0.13)	0.04 (0.10)	0.04 (0.12)	0.05 (0.11)
Proportion correct	Present	Present (*N* = 41)	0.27 (0.45)	0.68 (0.47)	0.47 (0.50)	0.57 (0.50)
		Absent (*N* = 32)	0.22 (0.41)	0.65 (0.48)	0.32 (0.47)	0.43 (0.50)
	Absent	Present (*N* = 36)	0.35 (0.48)	0.46 (0.50)	0.48 (0.50)	0.48 (0.50)
		Absent (*N* = 41)	0.32 (0.47)	0.34 (0.47)	0.33 (0.47)	0.37 (0.48)
Mean response time	Present	Present (*N*= 41)	14.44 (5.35)	11.65 (6.23)	17.21 (6.33)	15.60 (6.18)
		Absent (*N* = 32)	18.14 (5.63)	14.10 (6.09)	21.07 (6.38)	18.78 (6.27)
	Absent	Present (*N* = 36)	18.28 (6.76)	17.58 (6.74)	17.15 (7.42)	17.92 (6.74)
		Absent (*N* = 41)	18.54 (7.49)	20.11 (7.49)	20.07 (7.98)	18.36 (7.82)

**Fig. 3:**
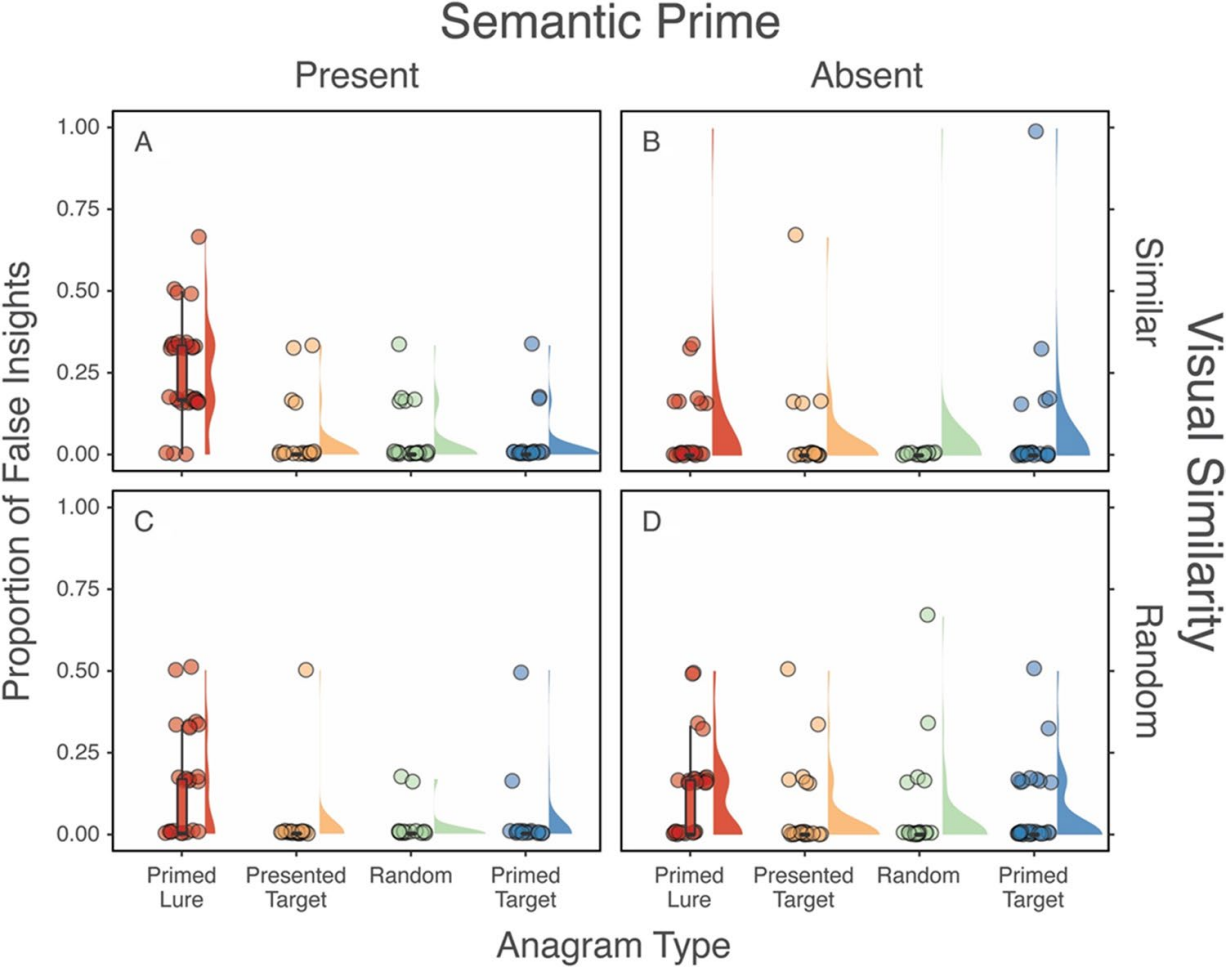
Percentage of trials with reported false insights for each anagram type in each condition

*Note.* The ‘raincloud’ plots in Fig. 3 depict the proportion of all trials with reported false insights across the four anagram types, combining boxplots, raw jittered data, and a split-half violin. Each plot represents one version of the experiment (between-subjects) and each distribution represents one type of anagram (within-subjects)

To test our first preregistered hypothesis, we examined the effects of the experimental manipulations on false insights by running a 2 (Semantic Priming: present, absent) 𝗑 2 (Visual Similarity: present, absent) 𝗑 4 (Anagram Type: primed lure, presented target, random, primed target) mixed ANOVA with Semantic Priming and Visual Similarity as the between-subjects factors and Anagram Type as the within-subjects factor. This analysis revealed the predicted main effect of Anagram Type, *F*(3,438) = 68.77, *p* < .001, η^2^_G_ = .11. To examine the source of this main effect of Anagram Type, we ran a series of post hoc Tukey comparisons. As can be seen in Fig. 3, these comparisons revealed a significant difference between the primed lure (*M =* 0.13, *SD* = 0.17) and each of the three other conditions: the presented target (*M* = 0.03, *SD =* 0.10), *t*(438) = 11.87, *p*<.001, *d* = 1.09, the primed target (*M =* 0.03, *SD* = 0.12), *t*(438) = 11.06, *p* < .001, *d* = 1.01, and the random anagrams (*M =* 0.03, *SD* = 0.11), *t*(438) = 12.15, *p* < .001, *d* = 1.06.

No main effect emerged for Visual Similarity, *F*(1,146) = 0.52, *p* = .474, η^2^_G_ < .01, or Semantic Priming, *F*(1,146) = 0.27, *p* = .606, η^2^_G_ < .01, and there was no interaction between these two variables, *F*(1,146) = 1.50, *p* = .223, η^2^_G_ < .01. An interaction emerged between Anagram Type and Semantic Priming, *F*(3,438) = 23.20, *p* < .001, η^2^_G_ = .04, and between Visual Similarity and Anagram Type, *F*(3,438) = 3.38 *p* = .018, η^2^_G_ < .01. Finally, a three-way interaction emerged between Anagram Type, Semantic Priming, and Visual Similarity, *F*(3,438) = 7.39, *p* < .001, η^2^_G_ = .01 (see Fig. 3).

### Exploratory analyses^3^

To decompose these interactions, we ran a series of Tukey pairwise comparisons between false insight rates for each anagram type across each level of Semantic Priming and Visual Similarity. These comparisons were almost exclusively significant when the lure was involved and almost exclusively not significant when the lure was not a target of comparison (for the results of all these comparisons see Tables 1–3 in the OSM). We therefore decided to run exploratory analyses on the lures to examine the joint effects of Semantic Priming and Visual Similarity. Specifically, we conducted a two-way, between-subjects ANOVA on false insight rates with the lure anagrams across both levels of Semantic Priming and Visual Similarity. This analysis revealed a main effect for Semantic Priming, with lures eliciting more false insights among participants who were exposed to priming (*M* = 0.21, *CI* = 0.17, 0.24) than those who were not (*M* = 0.07, *CI* = 0.03, 0.10), *F*(1,146) = 13.57, *p* < .001, η^2^_G_ = .09. No main effect emerged for Visual Similarity, *F*(1,146) = 3.09, *p* = .081, η^2^_G_ = .02, but a significant interaction emerged between Semantic Priming and Visual Similarity, *F*(1,146) = 7.17, *p* = .008, η^*2*^_G_ = .05.

To decompose this simple interaction effect, we first examined the effect of Visual Similarity at both levels of Semantic Priming. In the absence of Semantic Priming, there was no difference in false insight rates between visually similar (*M* = 0.07, *SE* = 0.03) and randomly scrambled (*M* = 0.09, *SE* = 0.03) anagrams, *t*(146) = 0.66, *p* = .510. In the presence of Semantic Priming, participants reported more false insights when the stimuli were visually similar (*M* = 0.24, *SE* = 0.02) than when they were randomly scrambled (*M* = 0.12, *SE* = 0.03), *t*(146) = -3.09, *p* = .002. Next, we examined the effect of priming at both levels of Visual Similarity. These analyses revealed that when anagrams were visually similar, Semantic Priming elicited significantly more false insights (*M =* 0.24, *SE* = 0.02) than no priming (*M =* 0.07, *SE* = 0.03), *t*(146) = -4.57, *p* < .001. When the anagrams were randomly scrambled, participants did not report more false insights for primed lures (*M =* 0.12, *SE* = 0.03) than those who received no Semantic Priming (*M =* 0.09, *SE* = 0.02), *t*(146) = -0.70, *p* = .485.

Next, we examined the false insight rates of the remaining three anagram types. A 2 (Semantic Priming: present, absent) 𝗑 2 (Visual Similarity: present, absent) 𝗑 3 (Anagram Type: presented target, primed target, random) ANOVA revealed no effect of Semantic Priming (Present: *M* = 0.02, Absent: *M* = 0.04), *F*(1,146) = 1.52, *p* = .220, η^2^_G_ = .01, Visual Similarity (Present: *M* = 0.03, Absent: *M* = 0.03), *F*(1,146) = 0.01, *p* = .941, η^2^_G_ < .01, or Anagram Type, *F*(2,292) = 1.27, *p* = .284, η^2^_G_ < .01, and no interaction between these three variables, *F*(2,292) = 0.75, *p* = .473, η^2^_G_ < .01. This analysis confirmed that the manipulations uniquely affected the primed lures and had virtually no impact on the remaining anagram types.

For our second preregistered analyses, as in Experiment 1, we examined whether participants gave weaker intensity ratings to false insights compared to correct ones. Again, we looked at false insights across all conditions and computed the mean intensity ratings for true and false insights for participants who experienced both (*N* = 76). A paired *t*-test revealed that false insights were again given lower intensity ratings (*M* = 5.43, *SD* = *2.18*) than correct ones (*M =* 6.06, *SD* = 1.88), *t*(75) *=* 3.03, *p* = .003, *d* = .35.

## General discussion

Across two experiments we demonstrated that false insights can be reliably induced through a combination of semantic priming and visual similarity. In Experiment 1, we found that participants experienced an overwhelming majority of false ‘Aha’ moments when solving anagrams that appeared similar to words for which they were semantically primed. In Experiment 2, we replicated this effect and found that both semantic priming and visual similarity were required to produce false insights. We also found that false insights for primed lures were positively associated with false memories for primed lures, consistent with the ‘discovery misattribution’ effect (Dougal Schooler, 2007). The results of both studies also confirmed prior findings that false insights are experientially weaker than true insights, which is again consistent with the findings of Danek and Wiley (2017).

The results of our studies provide a window into the origins of ‘Aha’ moments themselves, and answer questions about the informative value of ‘Aha’ phenomenology. According to the Eureka Heuristic framework (Laukkonen et al., 2018), insight phenomenology functions to ‘select’ ideas from the stream of consciousness by drawing attention to ideas that are most consistent with one’s implicit knowledge (Salvi et al., 2015, 2020). That is, ‘Aha’ moments operate as a heuristic – a mental shortcut for deciding which ideas to trust. Central to this view is the idea that feelings of insight are driven by past knowledge, and therefore if past knowledge is incorrect, then so too will be the insight (Laukkonen et al., 2020; Webb et al., 2019). In the above experiments, we manipulated past knowledge using the DRM paradigm in order to elicit *false* feelings of insight. These results thus highlight the fact that there is not a direct correspondence between insight and accuracy, and that the fidelity of insight phenomenology is crucially dependent on the knowledge that underlies them. This understanding has direct implications for our understanding of myriad false insights and their persuasive power in many domains, including fake news and misinformation, and the development of false or delusional beliefs.

Our findings are also broadly consistent with those of a recent study by Ammalainen and Moroshkina (2020), who found that the presentation of misleading pictorial hints could lead participants to a false anagram solution, and some of these false solutions appear to have been experienced as insight moments. Even though false insights were not the direct focus of their experiment, it is clear that their method could be used in the same manner as our own to isolate and study them. The positive correlation we found between false insights and false memories also supports previous findings that insight solutions are remembered more easily (Danek et al., 2013), and particularly suggests that feelings of ‘Aha’ can intrude upon a memory judgment such that one misattributes the feeling of discovery with the feeling of remembering (Dougal & Schooler, 2007).

### Limitations and future research

The two experiments reported here used anagrams, but of course there are dozens of problem types that have been and could be used for research on insight moments. Future work with other sorts of problems is needed to explore the potential of the DRM paradigm more broadly. For example, subsequent research could explore different underlying mechanisms of ‘Aha’ (e.g., restructuring, transfer, memory pops) that might lead to variable false insights in concert with the DRM paradigm. Relatedly, a somewhat open question remains as to *how* exactly participants arrived at their false insight: Did it follow an explicit or implicit process of inference? That is, did the false anagram solution appear to participants spontaneously (implicit route), or did they analytically infer that anagram solutions are sometimes associated with words from the list and thereby find a matching solution (explicit route). We favor the implicit route because previous research strongly indicates that ‘Aha’ moments tend to follow implicit processing (Amabile et al., 1986; Bowden, 1997; Grant & Spivey, 2003; Hattori et al., 2013; Laukkonen et al., 2020; Laukkonen et al., 2021; Maier, 1931; Salvi et al., 2015; Salvi & Bowden, 2020; Schunn & Dunbar, 1996; Sio & Ormerod, 2009). Since false anagram solutions had to be accompanied by ‘Aha’ experiences to be considered a ‘false insight,’ these false insights presumably occurred to the participant following implicit processing. Theoretically, if the explicit route was followed then ‘Aha’ moments would not have occurred in the first place.

We also had no way of equating the degree of visual similarity with the magnitude of sematic priming, so our conclusions regarding the joint importance of these two manipulations are limited to the current manipulations. Nonetheless, at this point we may tentatively conclude that false insights are a product of the same general processes as true insights, and thereby reflect a restructuring of a problem driven by past experience (Danek et al., 2020). Our results suggest that this sudden and dramatic restructuring can lead to false insights when the problem-solving context misleads people into inappropriately connecting ideas or experiences that do not actually belong together. In our paradigm, we lured people into this experience by planting an idea in their mind (semantic priming) and then providing a stimulus that closely resembled an instantiation of that idea (visual similarity), thereby making the solution seem feasible.

### Conclusions

Should we trust our epiphanies? Our research demonstrates that ‘Aha’ moments are not necessarily correct and that false insights can be induced by manipulating what one is thinking and seeing at the moment of solution. These results suggest that our feelings, while often informative, are also sometimes misleading. Like any other heuristic, feelings of insight are probably a useful guide due to their general accuracy but will occasionally lead us astray. Our experiments have established the importance of past information in the experience of false insight and provided a method to make the experimental study of false insights tractable.

### Supplementary Information

ESM 1 (DOCX 55 kb)

## Appendix 1

### Instructions transcript

You’ll begin by studying a list of words that will appear on the screen one by one. Your job is to remember as many of these words as possible. After seeing the word list, you will be asked to solve a few scrambled word problems. Simply do the best you can to try and unscramble these words into an English word as quickly as you can. Some may be quite difficult, so just go with whatever solution jumps out to you and don’t spend too long coming up with an answer, but just do your best to come up with a solution.

After you have typed your solution, we’re going to ask you how you arrived at your answer. In particular, we’re going to ask you whether you had an ‘insight experience’ or not. An ‘insight moment’ is when the solution to the problem suddenly becomes clear and obvious. Think of it as a miniature ‘Eureka’ moment or a ‘lightbulb’ moment. After solving each problem, please indicate whether you experienced an ‘insight moment’ or not. If you had an insight moment, we will ask you to rate how intense that feeling was, on a scale of 1–10. A rating of 1 on this scale means that you had a very weak insight and a rating of 10 means that you had a very strong insight. Again, it’s important that you enter your answer as soon as it comes to mind. Don’t try to work it out gradually.

After solving the anagrams, we’ll ask you to recall as many words as you can from the study list in the beginning. Type these words in the box provided then move on to the next set of words. This process will be repeated a few more times, and the whole experiment is designed to take about half an hour.

Next, we’re going to show you a practice trial of the task. This is just to show you how the experiment works so don’t worry too much about getting the answers right. Remember, you’ll see a list of words, solve some anagrams, recall as many words as you can, and tell us whether or not you had an insight moment! That’s all there is to it! The words will be presented automatically one by one, so there’s no need to click anything until the list has finished.

## Appendix 2

Stimulus sets for Experiment 1

Counterbalancing Condition^4^ A

**Table:**

			Study lists			
	Trial 1	Trial 2	Trial 3	Trial 4	Trial 5	Trial 6
Word 1	NERVOUS	REMEMBER	SAFE	PLANTS	SHOW	SCHOOL
Word 2	SCARED	SIGNIFICANT	SIMPLE	GRASS	EXAMPLE	UNIVERSITY
Word 3	GUILTY	HONOUR	SOFT	FARMER	PRESENT	TEACHER
Word 4	SHY	TRIBUTE	SECURE	SOIL	PROVE	LEARNING
Word 5	ANXIOUS	WASHINGTON	PURE	GLOVES	REVEAL	TRAINED
Word 6	UNCOMFORTABLE	MEMORIAL	INNOCENT	NURSERY	SUGGEST	TAUGHT
Word 7	ASHAMED	STATUE	GENTLE	COMPOST	TEACH	ORDERED
Word 8	SWEATY	ATTRACTION	COSY	WEEDS	DISPLAY	EXPLAINED
Word 9	HUMILIATED	MASTERPIECE	PEACEFUL	HOSE	CONFIRM	INFORMED
Word 10	BLUSH	MARKER	POWERLESS	SEEDLING	EXHIBITION	ENLIGHTENED
			Anagrams			
Primed lure	AMBASSADOR-ROAAMASBSD	MOMENTUM-MEMUNOMT	SHAMELESS-SSHMLEESA	ENDANGER-RGNDENEA	ADORNMENTS-SNMONETRAD	DESTRUCTION-NIOTTRUCSED
Presented target	HUMILIATED-DAMIUIHTEL	MEMORIAL-LAMORIME	POWERLESS-SSWERLPEO	SEEDLING- GLESDINE	EXHIBITION-BIXEHITONI	ENLIGHTENED-DENTGHIENEL
Primed target	DISTRAUGHT-TGSTRAUDHI	LANDMARK-KRMDNAAL	INNOCUOUS-SUNOCUONI	BOTANIST-TOBNATIS	ILLUSTRATE-ULLISTATRE	FAMILIARISE-ESAILIMRIFA
Random	MICROSCOPE-EIOPOSCMRC	COCKTAIL-LCOKIATC	HANDBRAKE-EKNDBRAHA	CLEANEST-SLENAECT	MOSQUITOES-SQUIMOTEOS	POMEGRANATE-ETMRGPANAOE

Counterbalancing Condition B

**Table:**

			Study lists			
	Trial 1	Trial 2	Trial 3	Trial 4	Trial 5	Trial 6
Word 1	INTERNATIONAL	POWER	CONFIDENT	RISK	JEWELLERY	DISASTER
Word 2	FOREIGN	ENERGY	LOUD	BRAVE	EMBELLISHMENT	RUIN
Word 3	AUTHORITY	DRIVE	EMBARRASSING	COMPROMISE	NECKLACE	DAMAGING
Word 4	EXECUTIVE	FORCE	BOLD	THREATEN	EARRINGS	DEVASTATION
Word 5	OFFICIAL	PUSH	CRUDE	HAZARD	RIBBON	MASSACRE
Word 6	AGENT	SPEED	OUTGOING	ABANDON	PENDANT	EXTINCTION
Word 7	POLITICIAN	VELOCITY	OBSCENE	DOOM	ACCESSORIES	HAVOC
Word 8	MINISTERIAL	DIRECTION	AUDACIOUS	VENTURE	ORNAMENTS	ELIMINATION
Word 9	REPRESENTATIVE	STRENGTH	IMPROPER	EXPOSE	ROSETTE	WRECKING
Word 10	EMBASSY	PACE	BRAZEN	MENACE	TINSEL	ABOLITION
			Anagrams			
Primed lure	EMBARRASSED-DEBASSRAMRE	MONUMENT-NEMOMTUN	HARMLESS- SSHMELRA	GARDENER-REEANGDR	DEMONSTRATE-SNMONETRAD	INSTRUCTED-DETTRUCISN
Presented target	MINISTERIAL-LIATSIERINM	VELOCITY-CELVOITY	OUTGOING-GUTNOIGO	THREATEN-ENRTATEH	ACCESSORIES-SEIESSORCCA	EXTINCTION-NXITCETION
Primed target	CONGRESSMAN-NAMGRESSOCN	MOVEMENT-NMVEOEMT	CAREFREE-EREFRAEC	FRIGHTEN-NEIGHTFR	DECORATIONS-SNCORATIDEO	DEMOLITION-NOMOLITIED
Random	PROPORTIONS-SRNROPTIOPO	WEEKENDS-SEEKENWD	VACATION-NOCATIAV	QUIZZING-GNIZZIQU	CONSISTENCY-YCNSISTENOC	MOTORCYCLE-EOTORLYCCM

Stimulus Sets for Experiment 2 ‘Both’ condition^5^

Stimulus Set A

**Table:**

			Study lists			
	Trial 1	Trial 2	Trial 3	Trial 4	Trial 5	Trial 6
Word 1	NERVOUS	REMEMBER	SAFE	PLANTS	TAKE	SCHOOL
Word 2	SCARED	SIGNIFICANT	SIMPLE	GRASS	COST	UNIVERSITY
Word 3	GUILTY	HONOUR	SOFT	FARMER	MINUS	TEACHER
Word 4	SHY	TRIBUTE	SECURE	SOIL	OPPOSED	LEARNING
Word 5	ANXIOUS	WASHINGTON	PURE	GLOVES	COUNTER	TRAINED
Word 6	UNCOMFORTABLE	MEMORIAL	INNOCENT	NURSERY	CONTRAST	TAUGHT
Word 7	ASHAMED	STATUE	GENTLE	COMPOST	UPSIDE	ORDERED
Word 8	SWEATY	ATTRACTION	COSY	WEEDS	VETO	EXPLAINED
Word 9	HUMILIATED	MASTERPIECE	PEACEFUL	HOSE	DOWNSIDE	INFORMED
Word 10	BLUSH	MARKER	POWERLESS	SEEDLING	UNWILLING	ENLIGHTENED
			Anagrams			
Primed lure	AMBASSADOR-ROAAMASBSD	MOMENTUM-MEMUNOMT	SHAMELESS-SSHMLEESA	ENDANGER-RGNDENEA	INVESTING- EGTINSIVN	DESTRUCTION-NIOTTRUCSED
Presented target	HUMILIATED-DAMIUIHTEL	MEMORIAL-LAMORIME	POWERLESS-SSWERLPEO	SEEDLING- GLESDINE	OPPOSED- EPPSOOS	ENLIGHTENED-DENTGHIENEL
Primed target	DISTRAUGHT-TGSTRAUDHI	LANDMARK-KRMDNAAL	INNOCUOUS-SUNOCUONI	BOTANIST-TOBNATIS	PESSIMIST- STMIEIPSS	FAMILIARISE-ESAILIMRIFA
Random	MICROSCOPE-EIOPOSCMRC	COCKTAIL-LCOKIATC	HANDBRAKE-EKNDBRAHA	CLEANEST-SLENAECT	SCRUBBING- SCURNGBBI	POMEGRANATE-ETMRGPANAOE

Stimulus Set B

**Table:**

			Study lists			
	Trial 1	Trial 2	Trial 3	Trial 4	Trial 5	Trial 6
Word 1	INTERNATIONAL	POWER	CONFIDENT	RISK	MONEY	DISASTER
Word 2	FOREIGN	ENERGY	LOUD	BRAVE	BUSINESS	RUIN
Word 3	AUTHORITY	DRIVE	EMBARRASSING	COMPROMISE	BUY	DAMAGING
Word 4	EXECUTIVE	FORCE	BOLD	THREATEN	SPEND	DEVASTATION
Word 5	OFFICIAL	PUSH	CRUDE	HAZARD	INTEREST	MASSACRE
Word 6	AGENT	SPEED	OUTGOING	ABANDON	SAVE	EXTINCTION
Word 7	POLITICIAN	VELOCITY	OBSCENE	DOOM	RISK	HAVOC
Word 8	MINISTERIAL	DIRECTION	AUDACIOUS	VENTURE	STOCKPILE	ELIMINATION
Word 9	REPRESENTATIVE	STRENGTH	IMPROPER	EXPOSE	CAPITAL	WRECKING
Word 10	EMBASSY	PACE	BRAZEN	MENACE	FUND	ABOLITION
			Anagrams			
Primed lure	EMBARRASSED-DEBASSRAMRE	MONUMENT-NEMOMTUN	HARMLESS- SSHMELRA	GARDENER-REEANGDR	NEGATIVES- ESTIVENAG	INSTRUCTED-DETTRUCISN
Presented target	MINISTERIAL-LIATSIERINM	VELOCITY-CELVOITY	OUTGOING-GUTNOIGO	THREATEN-ENRTATEH	BUSINESS- SNIBUSSE	EXTINCTION-NXITCETION
Primed target	CONGRESSMAN-NAMGRESSOCN	MOVEMENT-NMVEOEMT	CAREFREE-EREFRAEC	FRIGHTEN-NEIGHTFR	RESERVOIR- SEREROVIR	DEMOLITION-NOMOLITIED
Random	PROPORTIONS-SRNROPTIOPO	WEEKENDS-SEEKENWD	VACATION-NOCATIAV	QUIZZING-GNIZZIQU	PISTACHIO- HATSCIPIO	MOTORCYCLE-EOTORLYCCM

Stimulus Sets for Experiment 2 ‘No Priming’ condition

Stimulus Set A

**Table:**

			Study lists			
	Trial 1	Trial 2	Trial 3	Trial 4	Trial 5	Trial 6
Word 1	STIR	DISTORT	UNLIKE	REVOKE	ELEGANT	COAL
Word 2	PEPPER	FOLK	DISCIPLINE	BLOW	TRIVIAL	GLASS
Word 3	BIND	COMISSION	FEMININE	AGONY	BARK	REFORM
Word 4	INITIATIVE	FEAR	RESERVE	LARGE	DIFFICULT	TRUTH
Word 5	TUMBLE	GLORY	CORD	KNEEL	GLOVE	TERMINAL
Word 6	BEGINNING	REVISE	HAY	DOMINATE	SOAP	SERMON
Word 7	EXPECT	PATCH	MACHINERY	ALLOCATION	TACTIC	AUDIENCE
Word 8	GLOOM	COURTESY	VOYAGE	HEEL	SALVATION	COOPERATION
Word 9	FOOD	PANIC	SHAFT	FACTOR	STOCK	SEIZE
Word 10	GARAGE	DIAGRAM	ECONOMIC	FINISH	HIDE	MINDFUL
			Anagrams			
Primed lure	AMBASSADOR-ROAAMASBSD	MOMENTUM-MEMUNOMT	SHAMELESS-SSHMLEESA	ENDANGER-RGNDENEA	INVESTING- NGSNITVEI	DESTRUCTION-NIOTTRUCSED
Presented target	HUMILIATED-DAMIUIHTEL	MEMORIAL-LAMORIME	POWERLESS-SSWERLPEO	SEEDLING- GLESDINE	UNWILLING- GNLILWINU	ENLIGHTENED-DENTGHIENEL
Primed target	DISTRAUGHT-TGSTRAUDHI	LANDMARK-KRMDNAAL	INNOCUOUS-SUNOCUONI	BOTANIST-TOBNATIS	PESSIMIST- TESSIMIPS	FAMILIARISE-ESAILIMRIFA
Random	MICROSCOPE-EIOPOSCMRC	COCKTAIL-LCOKIATC	HANDBRAKE-EKNDBRAHA	CLEANEST-SLENAECT	SCRUBBING-GNRUBSIBC	POMEGRANATE-ETMRGPANAOE

Stimulus Set B

**Table:**

			Study lists			
	Trial 1	Trial 2	Trial 3	Trial 4	Trial 5	Trial 6
Word 1	RETIREMENT	STATEMENT	DISAPPOINTMENT	SWIPE	PARKING	PIERCE
Word 2	SLANT	DEAL	SUPPLY	VARIATION	DOUBLE	CHOOSE
Word 3	PROTECT	FEVER	ROAD	ADVOCATE	TIMBER	ENEMY
Word 4	TAPE	PREMIUM	THEME	FAILURE	CLEAN	MAZE
Word 5	JUST	CLOCK	BELONG	VISUAL	FORGE	DETAIL
Word 6	ROMANTIC	FORBID	GOD	AMBITION	WORKSHOP	ROOM
Word 7	RED	GLIMPSE	SIEGE	RESPONSIBILITY	RESTAURANT	MATHEMATICS
Word 8	PUBLISHER	BALD	THEORIST	THRONE	EXTINCT	PLANT
Word 9	WARNING	ACTION	CAPITAL	ORGANISE	COLLAR	ARM
Word 10	DISABILITY	SATELLITE	BUSINESS	WINDOW	WEAR	POWER
			Anagrams			
Primed lure	EMBARRASSED-DEBASSRAMRE	MONUMENT-NEMOMTUN	HARMLESS- SSHMELRA	GARDENER-REEANGDR	DEMONSTRATE-SNMONETRAD	INSTRUCTED-DETTRUCISN
Presented target	MINISTERIAL-LIATSIERINM	VELOCITY-CELVOITY	OUTGOING-GUTNOIGO	THREATEN-ENRTATEH	ACCESSORIES-SEIESSORCCA	EXTINCTION-NXITCETION
Primed target	CONGRESSMAN-NAMGRESSOCN	MOVEMENT-NMVEOEMT	CAREFREE-EREFRAEC	FRIGHTEN-NEIGHTFR	DECORATIONS-SNCORATIDEO	DEMOLITION-NOMOLITIED
Random	PROPORTIONS-SRNROPTIOPO	WEEKENDS-SEEKENWD	VACATION-NOCATIAV	QUIZZING-GNIZZIQU	CONSISTENCY-YCNSISTENOC	MOTORCYCLE-EOTORLYCCM

Stimulus Sets for No Visual Similarity Condition

Stimulus Set A

**Table:**

			Study lists			
	Trial 1	Trial 2	Trial 3	Trial 4	Trial 5	Trial 6
Word 1	NERVOUS	REMEMBER	SAFE	PLANTS	TAKE	SCHOOL
Word 2	SCARED	SIGNIFICANT	SIMPLE	GRASS	COST	UNIVERSITY
Word 3	GUILTY	HONOUR	SOFT	FARMER	MINUS	TEACHER
Word 4	SHY	TRIBUTE	SECURE	SOIL	OPPOSED	LEARNING
Word 5	ANXIOUS	WASHINGTON	PURE	GLOVES	COUNTER	TRAINED
Word 6	UNCOMFORTABLE	MEMORIAL	INNOCENT	NURSERY	CONTRAST	TAUGHT
Word 7	ASHAMED	STATUE	GENTLE	COMPOST	UPSIDE	ORDERED
Word 8	SWEATY	ATTRACTION	COSY	WEEDS	VETO	EXPLAINED
Word 9	HUMILIATED	MASTERPIECE	PEACEFUL	HOSE	DOWNSIDE	INFORMED
Word 10	BLUSH	MARKER	POWERLESS	SEEDLING	UNWILLING	ENLIGHTENED
			Anagrams			
Primed lure	AMBASSADOR-MAOADRSBAS	MOMENTUM-OMUMTNME	SHAMELESS-HASSEESML	ENDANGER-NDEEGNRA	INVESTING- EGTINSIVN	DESTRUCTION-ETIDTUNSORC
Presented target	HUMILIATED-IUHLAEIDTM	MEMORIAL-REMMOIAL	POWERLESS-OWSPERSEL	SEEDLING- DINELSEG	OPPOSED- EPPSOOS	ENLIGHTENED-NENDHIETGEL
Primed target	DISTRAUGHT-TGHTRIUDSA	LANDMARK-KLRMADNA	INNOCUOUS-NNUIOCSOU	BOTANIST-OTSBINTA	PESSIMIST- STMIEIPSS	FAMILIARISE-AIRFSLAIIME
Random	MICROSCOPE-IRPMOSECCO	COCKTAIL-LCCIOKAT	HANDBRAKE-ANKHABEDR	UNICORNS-NINUROSC	SCRUBBING- SCURNGBBI	POMEGRANATE-ORNPTGAEAME

Stimulus Set B

**Table:**

			Study lists			
	Trial 1	Trial 2	Trial 3	Trial 4	Trial 5	Trial 6
Word 1	INTERNATIONAL	POWER	CONFIDENT	RISK	MONEY	DISASTER
Word 2	FOREIGN	ENERGY	LOUD	BRAVE	BUSINESS	RUIN
Word 3	AUTHORITY	DRIVE	EMBARRASSING	COMPROMISE	BUY	DAMAGING
Word 4	EXECUTIVE	FORCE	BOLD	THREATEN	SPEND	DEVASTATION
Word 5	OFFICIAL	PUSH	CRUDE	HAZARD	INTEREST	MASSACRE
Word 6	AGENT	SPEED	OUTGOING	ABANDON	SAVE	EXTINCTION
Word 7	POLITICIAN	VELOCITY	OBSCENE	DOOM	RISK	HAVOC
Word 8	MINISTERIAL	DIRECTION	AUDACIOUS	VENTURE	STOCKPILE	ELIMINATION
Word 9	REPRESENTATIVE	STRENGTH	IMPROPER	EXPOSE	CAPITAL	WRECKING
Word 10	EMBASSY	PACE	BRAZEN	MENACE	FUND	ABOLITION
			Anagrams			
Primed lure	EMBARRASSED-ADARBERMSSE	MONUMENT-ETMOMNUN	HARMLESS- ALMSHERS	GARDENER-ERNEGRAD	NEGATIVES- ESTIVENAG	INSTRUCTED-RICEDSTUNT
Presented target	MINISTERIAL-ARINMELITSI	VELOCITY-EVYIOCTL	OUTGOING-GOGITONU	THREATEN-HERNTATE	BUSINESS- SNIBUSSE	EXTINCTION-CINENIXTOT
Primed target	CONGRESSMAN-AMENCRGSNOS	MOVEMENT-EMMETVON	CAREFREE-ERFAECRE	FRIGHTEN-EHFTGRIN	RESERVOIR- SEREROVIR	DEMOLITION-DIETLINOOM
Random	PROPORTIONS-PORISPORONT	WEEKENDS-KWNESSED	VACATION-TAOVINCA	QUIZZING-GIQZIUNZ	PISTACHIO- HATSCIPIO	MOTORCYCLE-CORTCOMLEY

Stimulus Sets for ‘Neither’ Condition

Stimulus Set A

**Table:**

			Study lists			
	Trial 1	Trial 2	Trial 3	Trial 4	Trial 5	Trial 6
Word 1	STIR	DISTORT	UNLIKE	REVOKE	ELEGANT	COAL
Word 2	PEPPER	FOLK	DISCIPLINE	BLOW	TRIVIAL	GLASS
Word 3	BIND	COMISSION	FEMININE	AGONY	BARK	REFORM
Word 4	INITIATIVE	FEAR	RESERVE	LARGE	DIFFICULT	TRUTH
Word 5	TUMBLE	GLORY	CORD	KNEEL	GLOVE	TERMINAL
Word 6	BEGINNING	REVISE	HAY	DOMINATE	SOAP	SERMON
Word 7	EXPECT	PATCH	MACHINERY	ALLOCATION	TACTIC	AUDIENCE
Word 8	GLOOM	COURTESY	VOYAGE	HEEL	SALVATION	COOPERATION
Word 9	FOOD	PANIC	SHAFT	FACTOR	STOCK	SEIZE
Word 10	GARAGE	DIAGRAM	ECONOMIC	FINISH	HIDE	MINDFUL
			Anagrams			
Primed lure	AMBASSADOR-MAOADRSBAS	MOMENTUM-OMUMTNME	SHAMELESS-HASSEESML	ENDANGER-NDEEGNRA	INVESTING- EGTINSIVN	DESTRUCTION-ETIDTUNSORC
Presented target	HUMILIATED-IUHLAEIDTM	MEMORIAL-REMMOIAL	POWERLESS-OWSPERSEL	SEEDLING- DINELSEG	OPPOSED- EPPSOOS	ENLIGHTENED-NENDHIETGEL
Primed target	DISTRAUGHT-TGHTRIUDSA	LANDMARK-KLRMADNA	INNOCUOUS-NNUIOCSOU	BOTANIST-OTSBINTA	PESSIMIST- STMIEIPSS	FAMILIARISE-AIRFSLAIIME
Random	MICROSCOPE-IRPMOSECCO	COCKTAIL-LCCIOKAT	HANDBRAKE-ANKHABEDR	CLEANEST- NCELASTE	SCRUBBING- SCURNGBBI	POMEGRANATE-ORNPTGAEAME

Stimulus Set B

**Table:**

			Study lists			
	Trial 1	Trial 2	Trial 3	Trial 4	Trial 5	Trial 6
Word 1	RETIREMENT	STATEMENT	DISAPPOINTMENT	SWIPE	PARKING	PIERCE
Word 2	SLANT	DEAL	SUPPLY	VARIATION	DOUBLE	CHOOSE
Word 3	PROTECT	FEVER	ROAD	ADVOCATE	TIMBER	ENEMY
Word 4	TAPE	PREMIUM	THEME	FAILURE	CLEAN	MAZE
Word 5	JUST	CLOCK	BELONG	VISUAL	FORGE	DETAIL
Word 6	ROMANTIC	FORBID	GOD	AMBITION	WORKSHOP	ROOM
Word 7	RED	GLIMPSE	SIEGE	RESPONSIBILITY	RESTAURANT	MATHEMATICS
Word 8	PUBLISHER	BALD	THEORIST	THRONE	EXTINCT	PLANT
Word 9	WARNING	ACTION	CAPITAL	ORGANISE	COLLAR	ARM
Word 10	DISABILITY	SATELLITE	BUSINESS	WINDOW	WEAR	POWER
			Anagrams			
Primed lure	EMBARRASSED-ADARBERMSSE	MONUMENT-ETMOMNUN	HARMLESS- ALMSHERS	GARDENER-ERNEGRAD	NEGATIVES- ESTIVENAG	INSTRUCTED-RICEDSTUNT
Presented target	MINISTERIAL-ARINMELITSI	VELOCITY-EVYIOCTL	OUTGOING-GOGITONU	THREATEN-HERNTATE	BUSINESS- SNIBUSSE	EXTINCTION-CINENIXTOT
Primed target	CONGRESSMAN-AMENCRGSNOS	MOVEMENT-EMMETVON	CAREFREE-ERFAECRE	FRIGHTEN-EHFTGRIN	RESERVOIR- SEREROVIR	DEMOLITION-DIETLINOOM
Random	PROPORTIONS-PORISPORONT	WEEKENDS-KWNESSED	VACATION-TAOVINCA	QUIZZING-GIQZIUNZ	PISTACHIO- HATSCIPIO	MOTORCYCLE-CORTCOMLEY

## Footnotes

1 In our preregistration, we referred to these anagrams with different labels. For clarity, we relabelled these anagrams to better communicate their relationship to the priming list.

2 In our preregistration, we specified that we intended to run a mixed-effects ANOVA with insight occurrence as a random effect. After collecting the data, we realized that our preregistration was made in error, and would not provide an appropriate test of our hypothesis. We instead ran a mixed ANOVA with anagram type as a within-participants factor, counterbalancing condition as a between-participants factor, and the proportion of trials for each anagram type that produced false insights as the dependent variable (Danek & Wiley, 2017).

3 In our preregistration, we specified analyses that were not the clearest way to test our focal hypotheses and follow up our significant interaction effects. The results of these analyses are consistent with predictions and can be found in the OSM, but we report more direct tests here.
