# Supplementary material for: Eliciting false insights with semantic priming
Source: Psychon Bull Rev. 2022 Feb 2;29(3):954–70. doi: 10.3758/s13423-021-02049-x (PMC9166882; doi:10.3758/s13423-021-02049-x)
Supplement: Supplementary file 1 — (DOCX 55 kb) [file 13423_2021_2049_MOESM1_ESM.docx]

**Experiment 1**

**Preregistered Analyses**

We predicted that participants would be faster to solve words presented on the study list (presented targets) than other types of anagrams. To test this hypothesis, we ran a repeated measures ANOVA with participants’ mean response times in seconds for each anagram type. This analysis revealed a significant effect of Anagram Type, *F*(3, 450) = 35.60, *p*<.001, *η^2^* = .09. To follow up this effect we conducted post-hoc Tukey comparisons. These comparisons revealed that presented targets (*M* = 152.35, *CI* = 132.37, 172.33) had significantly faster reaction times than the primed lures (*M* = 217.31, *CI* = 197.53, 237.65), *t*(447) = 6.32, *p* <.001). The presented targets were also solved significantly faster than the primed targets (*M* = 184.67, *CI* = 164.61, 204.74), *t*(447) = -3.15, *p* =.009), and the random anagrams (*M* = 256.00, *CI* =235.94, 276.06), *t*(447) = -10.01, *p* <.001).

**Experiment 2**

**Preregistered Analyses**

Our original preregistered analyses aimed to test the effect of Anagram Type at each experiment version. This analysis revealed that in the presence of both Semantic Priming and Visual Similarity, a significant effect of Anagram Type emerged as in Experiment 1, *F*(3, 160) = 61.10, *p*<.001, *η^2^* = .53 (see Figure 3A). A significant effect of Anagram Type also emerged in the presence of priming but absence of Visual Similarity, although the effect size was smaller, *F*(3, 124) = 22.40, *p*<.001, *η^2^* = .35 (see Figure 3B). Visual Similarity in the absence of priming yielded no effect for Anagram Type, *F*(3, 140) = 0.76, *p* = , *η^2^* = .02 (see Figure 3C), and no effect for Anagram Type emerged in the absence of Visual Similarity or Semantic Priming, *F*(3, 160) = 1.72, *p* = , *η^2^* = .03 (see Figure 3D).

**Table 1.**

*Pairwise Comparisons for the Interaction between Semantic Priming and Anagram Type*

| Comparison 1 | | Comparison 2 | | |  |  |  |  |  | |  |
| --- | --- | --- | --- | --- | --- | --- | --- | --- | --- | --- | --- |
| Semantic Priming | Anagram Type | Semantic Priming | Anagram Type | Estimate | SE | df | Lower CL | Upper CL | t.ratio | p.value | |
| Absent | Primed Lure | Present | Primed Lure | -0.10 | 0.03 | 146 | -0.18 | -0.02 | -3.68 | 0.008* | |
| Absent | Primed Lure | Absent | Presented Target | 0.05 | 0.01 | 146 | 0.00 | 0.09 | 3.24 | 0.032* | |
| Absent | Primed Lure | Present | Presented Target | 0.06 | 0.02 | 146 | -0.00 | 0.13 | 2.85 | 0.090 | |
| Absent | Primed Lure | Absent | Random | 0.05 | 0.01 | 146 | 0.00 | 0.09 | 3.39 | 0.012* | |
| Absent | Primed Lure | Present | Random | 0.06 | 0.02 | 146 | -0.00 | 0.13 | 2.86 | 0.089 | |
| Absent | Primed Lure | Absent | Primed Target | 0.03 | 0.01 | 146 | -0.01 | 0.08 | 2.11 | 0.411 | |
| Absent | Primed Lure | Present | Primed Target | 0.06 | 0.02 | 146 | -0.01 | 0.13 | 2.76 | 0.113 | |
| Present | Primed Lure | Absent | Presented Target | 0.14 | 0.02 | 146 | 0.08 | 0.21 | 6.55 | <.0001* | |
| Present | Primed Lure | Present | Presented Target | 0.16 | 0.02 | 146 | 0.11 | 0.20 | 10.52 | <.0001* | |
| Present | Primed Lure | Absent | Random | 0.15 | 0.02 | 146 | 0.08 | 0.22 | 6.42 | <.0001* | |
| Present | Primed Lure | Present | Random | 0.16 | 0.02 | 146 | 0.11 | 0.21 | 10.68 | <.0001* | |
| Present | Primed Lure | Absent | Primed Target | 0.13 | 0.02 | 146 | 0.06 | 0.20 | 5.56 | <.0001* | |
| Present | Primed Lure | Present | Primed Target | 0.16 | 0.02 | 146 | 0.11 | 0.21 | 10.31 | <.0001* | |
| Absent | Presented Target | Present | Presented Target | 0.01 | 0.02 | 146 | -0.04 | 0.06 | 0.90 | 0.986 | |
| Absent | Presented Target | Absent | Random | 0.00 | 0.01 | 146 | -0.02 | 0.03 | 0.28 | 1.000 | |
| Absent | Presented Target | Present | Random | 0.02 | 0.02 | 146 | -0.04 | 0.07 | 0.99 | 0.975 | |
| Absent | Presented Target | Absent | Primed Target | -0.02 | 0.01 | 146 | -0.04 | 0.01 | -1.75 | 0.658 | |
| Absent | Presented Target | Present | Primed Target | 0.02 | 0.02 | 146 | -0.04 | 0.07 | 0.90 | 0.986 | |
| Present | Presented Target | Absent | Random | -0.01 | 0.02 | 146 | -0.07 | 0.04 | -0.71 | 0.997 | |
| Present | Presented Target | Present | Random | 0.00 | 0.01 | 146 | -0.02 | 0.03 | 0.30 | 1.000 | |
| Present | Presented Target | Absent | Primed Target | -0.03 | 0.02 | 146 | -0.08 | 0.02 | -1.69 | 0.691 | |
| Present | Presented Target | Present | Primed Target | 0.00 | 0.01 | 146 | -0.03 | 0.03 | 0.16 | 1.000 | |
| Absent | Random | Present | Random | 0.01 | 0.02 | 146 | -0.04 | 0.07 | 0.81 | 0.992 | |
| Absent | Random | Absent | Primed Target | -0.02 | 0.01 | 146 | -0.04 | 0.01 | -2.09 | 0.428 | |
| Absent | Random | Present | Primed Target | 0.01 | 0.02 | 146 | -0.04 | 0.07 | 0.73 | 0.996 | |
| Present | Random | Absent | Primed Target | -0.03 | 0.02 | 146 | -0.09 | 0.02 | -1.74 | 0.663 | |
| Present | Random | Present | Primed Target | -0.00 | 0.01 | 146 | -0.03 | 0.03 | -0.13 | 1.000 | |
| Absent | Primed Target | Present | Primed Target | 0.03 | 0.02 | 146 | -0.03 | 0.09 | 1.64 | 0.727 | |

**Table 2.**

*Pairwise Comparisons for the Interaction between Visual Similarity and Anagram Type*

| Comparison 1 | | Comparison 2 | | |  |  | |  | |  |  |  |
| --- | --- | --- | --- | --- | --- | --- | --- | --- | --- | --- | --- | --- |
| Visual Similarity | Anagram Type | Visual Similarity | Anagram Type | Estimate | SE | df | Lower CL | | Upper CL | | t.ratio | p.value |
| Absent | Primed Lure | Present | Primed Lure | -0.05 | 0.03 | 146 | | -0.13 | | 0.03 | -1.76 | 0.649 |
| Absent | Primed Lure | Absent | Presented Target | 0.08 | 0.02 | 146 | | 0.03 | | 0.13 | 5.37 | <.0001* |
| Absent | Primed Lure | Present | Presented Target | 0.08 | 0.02 | 146 | | 0.01 | | 0.15 | 3.58 | 0.011* |
| Absent | Primed Lure | Absent | Random | 0.08 | 0.02 | 146 | | 0.04 | | 0.13 | 5.54 | <.0001* |
| Absent | Primed Lure | Present | Rando | 0.08 | 0.02 | 146 | | 0.01 | | 0.15 | 3.55 | 0.012* |
| Absent | Primed Lure | Absent | Primed Target | 0.07 | 0.02 | 146 | | 0.02 | | 0.12 | 4.65 | <.0001* |
| Absent | Primed Lure | Present | Primed Target | 0.07 | 0.02 | 146 | | 0.00 | | 0.14 | 3.18 | 0.038* |
| Present | Primed Lure | Absent | Presented Target | 0.13 | 0.02 | 146 | | 0.06 | | 0.19 | 5.87 | <.0001* |
| Present | Primed Lure | Present | Presented Target | 0.12 | 0.01 | 146 | | 0.08 | | 0.17 | 8.55 | <.0001* |
| Present | Primed Lure | Absent | Random | 0.13 | 0.02 | 146 | | 0.06 | | 0.20 | 5.76 | <.0001* |
| Present | Primed Lure | Present | Random | 0.13 | 0.01 | 146 | | 0.08 | | 0.17 | 8.70 | <.0001* |
| Present | Primed Lure | Absent | Primed Target | 0.12 | 0.02 | 146 | | 0.05 | | 0.19 | 5.17 | <.0001* |
| Present | Primed Lure | Present | Primed Target | 0.12 | 0.01 | 146 | | 0.07 | | 0.17 | 7.96 | <.0001* |
| Absent | Presented Target | Present | Presented Target | -0.00 | 0.02 | 146 | | -0.05 | | 0.05 | -0.14 | 1.000 |
| Absent | Presented Target | Absent | Random | 0.00 | 0.01 | 146 | | -0.02 | | 0.03 | 0.30 | 1.000 |
| Absent | Presented Target | Present | Random | 0.00 | 0.02 | 146 | | -0.05 | | 0.05 | 0.00 | 1.000 |
| Absent | Presented Target | Absent | Primed Target | -0.01 | 0.01 | 146 | | -0.04 | | 0.02 | -0.96 | 0.980 |
| Absent | Presented Target | Present | Primed Target | -0.01 | 0.02 | 146 | | -0.06 | | 0.05 | -0.43 | 1.000 |
| Present | Presented Target | Absent | Random | 0.00 | 0.02 | 146 | | -0.05 | | 0.06 | 0.28 | 1.000 |
| Present | Presented Target | Present | Random | 0.00 | 0.01 | 146 | | -0.02 | | 0.03 | 0.28 | 1.000 |
| Present | Presented Target | Absent | Primed Target | -0.01 | 0.02 | 146 | | -0.06 | | 0.05 | -0.36 | 1.000 |
| Present | Presented Target | Present | Primed Target | -0.01 | 0.01 | 146 | | -0.03 | | 0.02 | -0.59 | 0.999 |
| Absent | Random | Present | Random | -0.00 | 0.02 | 146 | | -0.06 | | 0.05 | -0.14 | 1.000 |
| Absent | Random | Absent | Primed Target | -0.01 | 0.01 | 146 | | -0.04 | | 0.02 | -1.29 | 0.900 |
| Absent | Random | Present | Primed Target | -0.01 | 0.02 | 146 | | -0.07 | | 0.05 | -0.54 | 0.999 |
| Present | Random | Absent | Primed Target | -0.01 | 0.02 | 146 | | -0.07 | | 0.05 | -0.47 | 1.000 |
| Present | Random | Present | Primed Target | -0.01 | 0.01 | 146 | | -0.03 | | 0.02 | -0.89 | 0.987 |
| Absent | Primed Target | Present | Primed Target | 0.00 | 0.02 | 146 | | -0.06 | | 0.06 | 0.06 | 1.000 |

**Table 3.**

*Pairwise Comparisons for the Interaction Between Semantic Priming* 𝗑 *Visual Similarity and Anagram Type*

| Comparison 1 | | | Comparison 2 | | | |  |  |  |  |  |  |  | |
| --- | --- | --- | --- | --- | --- | --- | --- | --- | --- | --- | --- | --- | --- | --- |
| Visual Similarity | Semantic Priming | Anagram Type | Visual Similarity | Semantic Priming | Anagram Type | | Estimate | SE | df | Lower CL | upper.CL | t.ratio | p.value |  |
| Present | Present | Presented Target | Present | Present | | Primed Target | 0.01 | 0.01 | 146 | -0.03 | 0.05 | 0.68 | 1.000 |  |
| Present | Present | Presented Target | Present | Present | | Random | 0.00 | 0.01 | 146 | -0.04 | 0.04 | 0.00 | 1.000 |  |
| Present | Present | Presented Target | Present | Absent | | Primed Target | -0.03 | 0.02 | 146 | -0.11 | 0.06 | -1.07 | 1.000 |  |
| Present | Present | Presented Target | Present | Absent | | Random | -0.00 | 0.02 | 146 | -0.09 | 0.08 | -0.14 | 1.000 |  |
| Present | Present | Presented Target | Absent | Present | | Primed Target | 0.00 | 0.03 | 146 | -0.09 | 0.09 | 0.14 | 1.000 |  |
| Present | Present | Presented Target | Absent | Present | | Random | 0.01 | 0.02 | 146 | -0.07 | 0.10 | 0.56 | 1.000 |  |
| Present | Present | Presented Target | Absent | Absent | | Primed Target | -0.02 | 0.02 | 146 | -0.11 | 0.06 | -1.03 | 1.000 |  |
| Present | Present | Presented Target | Absent | Absent | | Random | -0.01 | 0.02 | 146 | -0.09 | 0.07 | -0.53 | 1.000 |  |
| Present | Present | Primed Lure | Present | Present | | Presented Target | 0.21 | 0.02 | 146 | 0.14 | 0.28 | 10.64 | <.0001* |  |
| Present | Present | Primed Lure | Present | Present | | Primed Target | 0.22 | 0.02 | 146 | 0.15 | 0.29 | 10.73 | <.0001* |  |
| Present | Present | Primed Lure | Present | Present | | Random | 0.21 | 0.02 | 146 | 0.14 | 0.28 | 10.63 | <.0001* |  |
| Present | Present | Primed Lure | Present | Absent | | Presented Target | 0.20 | 0.03 | 146 | 0.10 | 0.31 | 6.84 | <.0001* |  |
| Present | Present | Primed Lure | Present | Absent | | Primed Target | 0.18 | 0.03 | 146 | 0.07 | 0.29 | 5.86 | <.0001* |  |
| Present | Present | Primed Lure | Present | Absent | | Random | 0.21 | 0.03 | 146 | 0.10 | 0.32 | 6.71 | <.0001* |  |
| Present | Present | Primed Lure | Absent | Present | | Presented Target | 0.22 | 0.03 | 146 | 0.11 | 0.33 | 7.26 | <.0001* |  |
| Present | Present | Primed Lure | Absent | Present | | Primed Target | 0.21 | 0.03 | 146 | 0.10 | 0.33 | 6.66 | <.0001* |  |
| Present | Present | Primed Lure | Absent | Present | | Random | 0.23 | 0.03 | 146 | 0.11 | 0.34 | 7.11 | <.0001* |  |
| Present | Present | Primed Lure | Absent | Absent | | Presented Target | 0.20 | 0.03 | 146 | 0.10 | 0.30 | 6.82 | <.0001* |  |
| Present | Present | Primed Lure | Absent | Absent | | Primed Target | 0.19 | 0.03 | 146 | 0.08 | 0.29 | 6.07 | <.0001* |  |
| Present | Present | Primed Lure | Absent | Absent | | Random | 0.20 | 0.03 | 146 | 0.09 | 0.31 | 6.57 | <.0001* |  |
| Present | Present | Random | Present | Present | | Primed Target | 0.01 | 0.01 | 146 | -0.03 | 0.05 | 0.70 | 1.000 |  |
| Present | Present | Random | Present | Absent | | Primed Target | -0.03 | 0.03 | 146 | -0.12 | 0.06 | -1.02 | 1.000 |  |
| Present | Present | Random | Absent | Present | | Primed Target | 0.00 | 0.03 | 146 | -0.09 | 0.10 | 0.13 | 1.000 |  |
| Present | Present | Random | Absent | Absent | | Primed Target | -0.02 | 0.03 | 146 | -0.11 | 0.06 | -0.97 | 1.000 |  |
| Present | Absent | Presented Target | Present | Present | | Presented Target | 0.01 | 0.02 | 146 | -0.07 | 0.09 | 0.36 | 1.000 |  |
| Present | Absent | Presented Target | Present | Present | | Primed Target | 0.02 | 0.02 | 146 | -0.07 | 0.10 | 0.66 | 1.000 |  |
| Present | Absent | Presented Target | Present | Present | | Random | 0.00 | 0.01 | 146 | -0.04 | 0.05 | 0.38 | 1.000 |  |
| Present | Absent | Presented Target | Present | Present | | Random | 0.01 | 0.02 | 146 | -0.08 | 0.09 | 0.34 | 1.000 |  |
| Present | Absent | Presented Target | Present | Absent | | Primed Target | -0.02 | 0.01 | 146 | -0.06 | 0.03 | -1.44 | 0.987 |  |
| Present | Absent | Presented Target | Absent | Present | | Presented Target | 0.02 | 0.02 | 146 | -0.07 | 0.10 | 0.71 | 1.000 |  |
| Present | Absent | Presented Target | Absent | Present | | Primed Target | 0.01 | 0.03 | 146 | -0.08 | 0.10 | 0.44 | 1.000 |  |
| Present | Absent | Presented Target | Absent | Present | | Random | 0.02 | 0.03 | 146 | -0.07 | 0.11 | 0.86 | 1.000 |  |
| Present | Absent | Presented Target | Absent | Absent | | Primed Target | -0.02 | 0.02 | 146 | -0.10 | 0.07 | -0.67 | 1.000 |  |
| Present | Absent | Presented Target | Absent | Absent | | Random | -0.00 | 0.02 | 146 | -0.09 | 0.08 | -0.18 | 1.000 |  |
| Present | Absent | Primed Lure | Present | Present | | Presented Target | 0.05 | 0.03 | 146 | -0.06 | 0.15 | 1.47 | 0.984 |  |
| Present | Absent | Primed Lure | Present | Present | | Primed Lure | -0.17 | 0.04 | 146 | -0.29 | -0.04 | -4.57 | 0.001* |  |
| Present | Absent | Primed Lure | Present | Present | | Primed Target | 0.05 | 0.03 | 146 | -0.06 | 0.17 | 1.65 | 0.955 |  |
| Present | Absent | Primed Lure | Present | Present | | Random | 0.05 | 0.03 | 146 | -0.07 | 0.16 | 1.42 | 0.989 |  |
| Present | Absent | Primed Lure | Present | Absent | | Presented Target | 0.04 | 0.02 | 146 | -0.04 | 0.11 | 1.75 | 0.930 |  |
| Present | Absent | Primed Lure | Present | Absent | | Primed Target | 0.02 | 0.02 | 146 | -0.06 | 0.09 | 0.85 | 1.000 |  |
| Present | Absent | Primed Lure | Present | Absent | | Random | 0.04 | 0.02 | 146 | -0.03 | 0.12 | 1.96 | 0.841 |  |
| Present | Absent | Primed Lure | Absent | Present | | Presented Target | 0.05 | 0.03 | 146 | -0.06 | 0.16 | 1.70 | 0.944 |  |
| Present | Absent | Primed Lure | Absent | Present | | Primed Lure | -0.05 | 0.04 | 146 | -0.19 | 0.08 | -1.30 | 0.995 |  |
| Present | Absent | Primed Lure | Absent | Present | | Primed Target | 0.05 | 0.03 | 146 | -0.07 | 0.17 | 1.45 | 0.986 |  |
| Present | Absent | Primed Lure | Absent | Present | | Random | 0.06 | 0.03 | 146 | -0.06 | 0.17 | 1.79 | 0.917 |  |
| Present | Absent | Primed Lure | Absent | Absent | | Presented Target | 0.03 | 0.03 | 146 | -0.07 | 0.14 | 1.07 | 1.000 |  |
| Present | Absent | Primed Lure | Absent | Absent | | Primed Target | 0.02 | 0.03 | 146 | -0.09 | 0.13 | 0.64 | 1.000 |  |
| Present | Absent | Primed Lure | Absent | Absent | | Random | 0.03 | 0.03 | 146 | -0.08 | 0.14 | 1.04 | 1.000 |  |
| Present | Absent | Primed Target | Present | Present | | Primed Target | 0.03 | 0.03 | 146 | -0.06 | 0.13 | 1.30 | 0.995 |  |
| Present | Absent | Primed Target | Absent | Present | | Primed Target | 0.03 | 0.03 | 146 | -0.07 | 0.13 | 1.06 | 1.000 |  |
| Present | Absent | Random | Present | Present | | Primed Target | 0.01 | 0.03 | 146 | -0.08 | 0.10 | 0.44 | 1.000 |  |
| Present | Absent | Random | Present | Present | | Random | 0.00 | 0.03 | 146 | -0.09 | 0.09 | 0.13 | 1.000 |  |
| Present | Absent | Random | Present | Absent | | Primed Target | -0.02 | 0.01 | 146 | -0.07 | 0.02 | -1.87 | 0.883 |  |
| Present | Absent | Random | Absent | Present | | Random | 0.02 | 0.03 | 146 | -0.08 | 0.11 | 0.64 | 1.000 |  |
| Present | Absent | Random | Absent | Absent | | Primed Target | -0.02 | 0.03 | 146 | -0.11 | 0.07 | -0.81 | 1.000 |  |
| Present | Absent | Random | Absent | Absent | | Primed Target | 0.01 | 0.03 | 146 | -0.09 | 0.10 | 0.25 | 1.000 |  |
| Absent | Present | Presented Target | Present | Present | | Primed Target | -0.04 | 0.03 | 146 | -0.13 | 0.06 | -1.36 | 0.993 |  |
| Absent | Present | Presented Target | Present | Present | | Primed Target | -0.01 | 0.02 | 146 | -0.09 | 0.07 | -0.38 | 1.000 |  |
| Absent | Present | Presented Target | Present | Present | | Primed Target | -0.00 | 0.03 | 146 | -0.09 | 0.09 | -0.03 | 1.000 |  |
| Absent | Present | Presented Target | Present | Present | | Random | -0.01 | 0.02 | 146 | -0.09 | 0.08 | -0.36 | 1.000 |  |
| Absent | Present | Presented Target | Present | Absent | | Random | -0.01 | 0.03 | 146 | -0.10 | 0.08 | -0.48 | 1.000 |  |
| Absent | Present | Presented Target | Absent | Present | | Random | 0.01 | 0.01 | 146 | -0.04 | 0.05 | 0.40 | 1.000 |  |
| Absent | Present | Presented Target | Absent | Absent | | Primed Target | -0.03 | 0.03 | 146 | -0.12 | 0.05 | -1.32 | 0.995 |  |
| Absent | Present | Presented Target | Absent | Absent | | Primed Target | -0.01 | 0.01 | 146 | -0.05 | 0.04 | -0.38 | 1.000 |  |
| Absent | Present | Presented Target | Absent | Absent | | Random | -0.02 | 0.02 | 146 | -0.11 | 0.06 | -0.85 | 1.000 |  |
| Absent | Present | Primed Lure | Present | Present | | Presented Target | 0.10 | 0.03 | 146 | -0.02 | 0.21 | 2.98 | 0.192 |  |
| Absent | Present | Primed Lure | Present | Present | | Primed Lure | -0.12 | 0.04 | 146 | -0.25 | 0.02 | -3.09 | 0.148 |  |
| Absent | Present | Primed Lure | Present | Present | | Primed Target | 0.10 | 0.03 | 146 | -0.01 | 0.22 | 3.09 | 0.147 |  |
| Absent | Present | Primed Lure | Present | Present | | Random | 0.10 | 0.03 | 146 | -0.02 | 0.21 | 2.88 | 0.236 |  |
| Absent | Present | Primed Lure | Present | Absent | | Presented Target | 0.09 | 0.03 | 146 | -0.03 | 0.20 | 2.69 | 0.351 |  |
| Absent | Present | Primed Lure | Present | Absent | | Primed Target | 0.07 | 0.03 | 146 | -0.05 | 0.19 | 2.01 | 0.813 |  |
| Absent | Present | Primed Lure | Present | Absent | | Random | 0.09 | 0.03 | 146 | -0.03 | 0.21 | 2.73 | 0.322 |  |
| Absent | Present | Primed Lure | Absent | Present | | Presented Target | 0.10 | 0.02 | 146 | 0.03 | 0.18 | 4.63 | 0.001* |  |
| Absent | Present | Primed Lure | Absent | Present | | Primed Target | 0.10 | 0.02 | 146 | 0.02 | 0.18 | 4.27 | 0.004* |  |
| Absent | Present | Primed Lure | Absent | Present | | Random | 0.11 | 0.02 | 146 | 0.03 | 0.19 | 4.86 | 0.0003* |  |
| Absent | Present | Primed Lure | Absent | Absent | | Presented Target | 0.08 | 0.03 | 146 | -0.03 | 0.19 | 2.60 | 0.409 |  |
| Absent | Present | Primed Lure | Absent | Absent | | Primed Target | 0.07 | 0.03 | 146 | -0.05 | 0.19 | 2.12 | 0.750 |  |
| Absent | Present | Primed Lure | Absent | Absent | | Random | 0.08 | 0.03 | 146 | -0.03 | 0.20 | 2.52 | 0.466 |  |
| Absent | Present | Primed Target | Present | Present | | Primed Target | 0.00 | 0.03 | 146 | -0.09 | 0.10 | 0.17 | 1.000 |  |
| Absent | Present | Random | Present | Present | | Primed Target | -0.01 | 0.03 | 146 | -0.10 | 0.09 | -0.22 | 1.000 |  |
| Absent | Present | Random | Present | Present | | Random | -0.01 | 0.03 | 146 | -0.11 | 0.08 | -0.53 | 1.000 |  |
| Absent | Present | Random | Present | Absent | | Primed Target | -0.04 | 0.03 | 146 | -0.14 | 0.06 | -1.47 | 0.985 |  |
| Absent | Present | Random | Absent | Present | | Primed Target | -0.01 | 0.01 | 146 | -0.06 | 0.04 | -0.80 | 1.000 |  |
| Absent | Present | Random | Absent | Absent | | Primed Target | -0.04 | 0.03 | 146 | -0.13 | 0.06 | -1.43 | 0.988 |  |
| Absent | Absent | Presented Target | Present | Present | | Presented Target | 0.01 | 0.02 | 146 | -0.06 | 0.09 | 0.56 | 1.000 |  |
| Absent | Absent | Presented Target | Present | Present | | Primed Target | 0.02 | 0.02 | 146 | -0.06 | 0.10 | 0.86 | 1.000 |  |
| Absent | Absent | Presented Target | Present | Present | | Random | 0.01 | 0.02 | 146 | -0.07 | 0.09 | 0.53 | 1.000 |  |
| Absent | Absent | Presented Target | Present | Absent | | Presented Target | 0.00 | 0.02 | 146 | -0.07 | 0.08 | 0.19 | 1.000 |  |
| Absent | Absent | Presented Target | Present | Absent | | Primed Target | -0.01 | 0.02 | 146 | -0.10 | 0.07 | -0.58 | 1.000 |  |
| Absent | Absent | Presented Target | Present | Absent | | Random | 0.01 | 0.02 | 146 | -0.08 | 0.09 | 0.37 | 1.000 |  |
| Absent | Absent | Presented Target | Absent | Present | | Presented Target | 0.02 | 0.02 | 146 | -0.06 | 0.10 | 0.91 | 1.000 |  |
| Absent | Absent | Presented Target | Absent | Present | | Primed Target | 0.02 | 0.03 | 146 | -0.07 | 0.11 | 0.61 | 1.000 |  |
| Absent | Absent | Presented Target | Absent | Present | | Random | 0.03 | 0.02 | 146 | -0.06 | 0.11 | 1.05 | 1.000 |  |
| Absent | Absent | Presented Target | Absent | Absent | | Primed Target | -0.01 | 0.01 | 146 | -0.05 | 0.03 | -1.01 | 1.000 |  |
| Absent | Absent | Presented Target | Absent | Absent | | Random | 0.00 | 0.01 | 146 | -0.04 | 0.04 | 0.00 | 1.000 |  |
| Absent | Absent | Primed Lure | Present | Present | | Presented Target | 0.07 | 0.03 | 146 | -0.03 | 0.17 | 2.37 | 0.576 |  |
| Absent | Absent | Primed Lure | Present | Present | | Primed Lure | -0.14 | 0.04 | 146 | -0.26 | -0.02 | -4.05 | 0.008* |  |
| Absent | Absent | Primed Lure | Present | Present | | Primed Target | 0.08 | 0.03 | 146 | -0.03 | 0.18 | 2.51 | 0.472 |  |
| Absent | Absent | Primed Lure | Present | Present | | Random | 0.07 | 0.03 | 146 | -0.04 | 0.17 | 2.28 | 0.640 |  |
| Absent | Absent | Primed Lure | Present | Absent | | Presented Target | 0.06 | 0.03 | 146 | -0.04 | 0.16 | 2.05 | 0.791 |  |
| Absent | Absent | Primed Lure | Present | Absent | | Primed Lure | 0.02 | 0.04 | 146 | -0.10 | 0.15 | 0.66 | 1.000 |  |
| Absent | Absent | Primed Lure | Present | Absent | | Primed Target | 0.04 | 0.03 | 146 | -0.07 | 0.15 | 1.35 | 0.993 |  |
| Absent | Absent | Primed Lure | Present | Absent | | Random | 0.07 | 0.03 | 146 | -0.04 | 0.17 | 2.12 | 0.750 |  |
| Absent | Absent | Primed Lure | Absent | Present | | Presented Target | 0.08 | 0.03 | 146 | -0.03 | 0.18 | 2.57 | 0.428 |  |
| Absent | Absent | Primed Lure | Absent | Present | | Primed Lure | -0.03 | 0.04 | 146 | -0.16 | 0.10 | -0.70 | 1.000 |  |
| Absent | Absent | Primed Lure | Absent | Present | | Primed Target | 0.07 | 0.03 | 146 | -0.04 | 0.19 | 2.25 | 0.660 |  |
| Absent | Absent | Primed Lure | Absent | Present | | Random | 0.08 | 0.03 | 146 | -0.03 | 0.19 | 2.62 | 0.393 |  |
| Absent | Absent | Primed Lure | Absent | Absent | | Presented Target | 0.06 | 0.02 | 146 | -0.01 | 0.13 | 2.87 | 0.246 |  |
| Absent | Absent | Primed Lure | Absent | Absent | | Primed Target | 0.04 | 0.02 | 146 | -0.03 | 0.12 | 2.19 | 0.706 |  |
| Absent | Absent | Primed Lure | Absent | Absent | | Random | 0.06 | 0.02 | 146 | -0.01 | 0.13 | 2.86 | 0.248 |  |
| Absent | Absent | Primed Target | Present | Present | | Primed Target | 0.03 | 0.03 | 146 | -0.06 | 0.12 | 1.27 | 0.997 |  |
| Absent | Absent | Primed Target | Present | Absent | | Primed Target | -0.00 | 0.03 | 146 | -0.09 | 0.09 | -0.08 | 1.000 |  |
| Absent | Absent | Primed Target | Absent | Present | | Primed Target | 0.03 | 0.03 | 146 | -0.07 | 0.12 | 1.02 | 1.000 |  |
| Absent | Absent | Random | Present | Present | | Primed Target | 0.02 | 0.03 | 146 | -0.07 | 0.11 | 0.81 | 1.000 |  |
| Absent | Absent | Random | Present | Present | | Random | 0.01 | 0.02 | 146 | -0.07 | 0.10 | 0.50 | 1.000 |  |
| Absent | Absent | Random | Present | Absent | | Primed Target | -0.01 | 0.03 | 146 | -0.11 | 0.08 | -0.55 | 1.000 |  |
| Absent | Absent | Random | Present | Absent | | Random | 0.01 | 0.03 | 146 | -0.08 | 0.10 | 0.35 | 1.000 |  |
| Absent | Absent | Random | Absent | Present | | Primed Target | 0.02 | 0.03 | 146 | -0.08 | 0.11 | 0.59 | 1.000 |  |
| Absent | Absent | Random | Absent | Present | | Random | 0.03 | 0.03 | 146 | -0.07 | 0.12 | 1.00 | 1.000 |  |
| Absent | Absent | Random | Absent | Absent | | Primed Target | -0.01 | 0.01 | 146 | -0.05 | 0.03 | -1.05 | 1.000 |  |
